# Risk factors associated with readmissions of patients with severe mental disorders under treatment with antipsychotics

**DOI:** 10.1186/s12888-022-03794-6

**Published:** 2022-03-17

**Authors:** Ronaldo Portela, Milton Leonard Wainberg, Saulo Castel, Helian Nunes de Oliveira, Cristina Mariano Ruas

**Affiliations:** 1 Faculty of Pharmacy, Social Pharmacy Department, UFMG, PPGMAF, Presidente Antônio Carlos, Av., 6627 - Pampulha CEP: 31270-901, Belo Horizonte MG, Brasil; 2 Department of Psychiatry, Columbia University, New York State Psychiatric Institute, New York, USA; 3 Department of Psychiatry, Sunnybrook Health Sciences Centre, University of Toronto, Toronto, Canada; 4 UFMG, Social and Preventive Medicine Department of Medical School, Belo Horizonte, Brazil

**Keywords:** Severe mental disorders, Antipsychotics, Readmission, Psychiatric Hospital

## Abstract

**Background:**

The aim of this study was to assess the risk of readmission in patients with severe mental disorders, compare it between patients using different types of antipsychotics and determine risk factors for psychiatric readmission.

**Methods:**

Medical records of a non-concurrent cohort of 625 patients with severe mental disorders (such as psychoses and severe mood disorders) who were first discharged from January to December 2012 (entry into the cohort), with longitudinal follow-up until December 2017 constitute the sample. Descriptive statistical analysis of characteristics of study sample was performed. The risk factors for readmission were assessed using Cox regression.

**Results:**

Males represented 51.5% of the cohort, and 75.6% of the patients had no partner. Most patients (89.9%) lived with relatives, and 64.7% did not complete elementary school. Only 17.1% used more than one antipsychotic, 34.2% did not adhere to the treatment, and 13.9% discontinued the medication due to unavailability in public pharmacies. There was a need to change the antipsychotic due to the lack of therapeutic response (11.2% of the patients) and adverse reactions to the antipsychotic (5.3% of the patients). Cox regression showed that the risk of readmission was increased by 25.0% (RR, 1.25; 95% CI, 1.03–1.52) when used typical antipsychotics, compared to those who used atypical ones, and by 92.0% (RR, 1.92; 95% CI, 1.63–2.27) when patients did not adhere to maintenance treatment compared to those who adhered.

**Conclusions:**

Use of atypical antipsychotics and adherence to treatment were associated with a lower risk of psychiatric readmissions.

## Background

Unlike common mental disorders, the National Institute of Mental Health (NIMH) defines a severe mental disorder as a mental, behavioral, or emotional disorder that results in severe functional impairment, which substantially interferes with or limits one or more life activities [1]. These diseases include psychoses (mainly schizophrenia and bipolar affective disorder) and severe mood disorders characterized by long-term treatment, lasting 2 years or more and profound disability in social and occupational performance and daily activities, if left untreated [2–6].

As there are no specific biomarkers to diagnose or characterize the severity of mental disorders, psychiatric disorder epiphenomena should be assessed to determine their severity with severity criteria established by the World Health Organization (WHO) through the International Classification of Diseases (ICD-10), maintained in the 11^th^ edition (ICD-11) scheduled to enter into force in January 2022, or by the American Psychiatric Association through the Diagnostic and Statistical Manual of Mental Disorders (DSM-5) [7–10].

According to WHO, based on analysis by the 2017 Global Burden of Disease Study, among severe mental disorders worldwide, bipolar affective disorder and schizophrenia affect 45 million and 20 million people, respectively [7, 11]. In 2017, NIMH reported 11.2 million adults over 18 in the United States with a severe mental disorder, representing 4.5% of all adults in the United States – 5.7% among females and 3.3% among males [1, 7]. Brazil has not yet produced representative studies on the prevalence rates of individuals with severe mental disorders. However, in 2005, per the Brazilian Ministry of Health, approximately 5 million people, about 3% of all adults, required continuous mental health care due to severe mental disorders such as psychosis severe mood disorders [12, 13].

Antipsychotics are used to treat patients with severe mental disorders to reduce the frequency and severity of psychotic outbreaks that lead to the need for readmissions and other symptoms, thus improving functional capacity and quality of life, and psychosocial interventions are complementary to pharmacological treatment [14, 15]. Antipsychotics are classified into two major groups: typical and atypical. Their adverse reaction profiles can be severe, including extrapyramidal symptoms (dystonia, akathisia, and parkinsonism, which occur more acutely, and more chronic manifestations of tardive akathisia and tardive dyskinesia) and metabolic changes (weight gain and type 2 diabetes), for typical antipsychotics and that can occur with the use of atypical antipsychotics, respectively [16]. Despite mostly similar efficacy to typical antipsychotics [17–20], where available, atypical antipsychotics have been elected as the first choice in treating severe mental disorders [4, 21].

Psychiatric readmission is related to multiple factors, which transcend the mental disorder severity. Antipsychotic pharmacotherapy should be evaluated, mainly under the tolerability spectrum, as a potential factor associated with the risk of readmissions [22]. Rehospitalization may also be associated with patients’ social determinants of health, access to quality outpatient care and medication, and adherence to treatment [23–25].

### Aims and objectives

This study evaluated risk factors associated with readmissions in a cohort of patients diagnosed with a severe mental disorder followed for 5 years.

## Methods

### Study design

Non-concurrent cohort study with 625 patients with severe mental disorders (psychoses and severe mood disorders) discharged from January to December 2012 (cohort entry period), with longitudinal follow-up until December 2017 in a psychiatric hospital in the public health network of Brasília, Federal District, Brazil.

### Population and sampling

A total of 1,273 patients with severe mental disorders had a first discharge record from the follow-up period during the cohort entry period (January 1 to December 31, 2012).

For sample selection, the numbers of the electronic medical records were randomized by the Microsoft Excel® program using the Sort Range Randomly tool to allow all patients to have the opportunity to be selected for the study. The 60% readmission proportion was considered to calculate the sample size, which is the mean readmission rate described in the literature [26, 27], and the methodology for estimating proportions for finite populations [28] was used. The sample calculation function of the R® software was used for this purpose.

The study included patients with a severe mental disorder hospitalized for disorders with recurrent psychotic episodes, such as schizophrenia, bipolar affective disorders, persistent delusional disorders, schizoaffective disorders, and other psychoses, first discharged from January 1 to December 31, 2012 (discharge from hospitalization index of the study), and who continued to be followed up until December 31, 2017.

Patients who continued maintenance treatment (out-of-hospital) in outpatient services in other states in the country and those without any data or records in electronic medical records were excluded from the study.

A total of 625 medical records were selected to the study the patients who received their first hospital discharge from January 1 to December 31, 2012, and who continued the followed up by the hospital outpatient clinic.

### Statistical analysis

Descriptive statistical analyses and absolute and relative frequencies were adopted to evaluate the sociodemographic and clinical characteristics and the monotherapy used in the maintenance treatment. Sociodemographic characteristics were extracted from the patient’s record, and clinical characteristics such as adherence to treatment, presence of lifetime suicide attempt, substance use, and antipsychotic pharmacotherapy were obtained by reviewing medical reports found in the medical records.

Univariate and multivariate Cox regressions were performed to test the associations between readmissions during the study period and the variables related to patients who used antipsychotic monotherapy until the end of the observation. In regressions, the initial time was the date of discharge from the first hospitalization (index hospitalization of this study), and the failure time was the number of days between new hospitalizations. Cox regression was selected because the event is used as a response variable, which is readmission or not, and the time of failure, which would enter the model as an explanatory variable in logistic regression.

A stepwise method was implemented to select the variables. This method is defined as a mix of backward and forward [29] methods. For the multivariate analysis, variables with a *p*-value of less than 0.25 were selected from the univariate analysis using the forward method [29].

The backward method was applied for the multivariate analysis. This method consists of removing, one at a time, the variable with the highest *p*-value, repeating the procedure until only significant variables are left in the model [29]. A 5% level of significance was adopted, and variables with a *p*-value slightly above that value were also accepted, which were considered marginally significant.

The Cox model’s assumption of proportional risks was verified using the risk proportionality test [30], considering a significance level of 5%. The Relative Risk (RR) was used as a measure of effect based on the reason for readmission. R® version 3.6.1 (R Foundation for Statistical Computing) was the software used in the analysis.

### Ethical considerations

The study was approved by the Research Ethics Committee (COEP) of the Health Sciences Teaching and Research Foundation of the State Health Department of the Federal District (FEPECS-SESDF) under Opinion N° 2.138.356.

The consent form was dispensed as this study was based on information from medical records collected without the patients’ nominal identification.

## Results

Approximately 51.5% of the 625 patients were male, and 75.6% had no partner. Most patients, 89.9%, lived with relatives, and 64.7% did not complete elementary school. Most, 36.8%, never worked or were unemployed, 35.1, and 8.5% left work because of the severe mental disorder. The rate of lifetime suicide attempts among the 625 patients was 11.0%. Approximately 50.8% of the patients who used licit substances used tobacco, while 39.9% used alcohol, and 29.2% of them used both, while 28.8% patients who used illicit substances used marijuana, 27.8% used cocaine, and 19.2% used both (Table 1).

**Table 1:** Sociodemographic and clinical profile of patients followed-up from 2012 to 2017, Brasília—Brazil

Characteristic	Patients, No. (%)
	**All patients** **(*N* = 625)**
Sex	
Male	322 (51.5)
Female	303 (48.5)
Marital status (*n* = 528)	
No partner	399 (75.6)
With partner	129 (24.4)
Housing circumstances (*n* = 576)	
Lives with family members	518 (89.9)
Lives alone	19 (3.2)
Lives in a public hostel	17 (3.0)
Homeless	17 (3.0)
Other	5 (0.9)
Schooling (*n* = 495)	
Illiterate	320 (64.7)
Elementary	71 (14.3)
High school	91 (18.4)
Higher education	13 (2.6)
Occupation (*n* = 402)	
Never worked	148 (36.8)
Unemployed	141 (35.1)
Employee/Regular activity	68 (16.9)
Retired due to disease	34 (8.5)
Retired for working time	11 (2.7)
**Lifetime suicide attempt**	
Yes	69 (11.0)
No	556 (89.0)
Licit substances users (*n* = 514)	
None	198 (38.5)
Tobacco only	111 (21.6)
Alcohol only	55 (10.7)
Both	150 (29.2)
Illicit substances users (*n* = 364)	
None	228 (62.6)
Cannabis only	35 (9.6)
Cocaine only	31 (8.6)
Both	70 (19.2)

Most patients, 82.9%, only took one antipsychotic in their maintenance treatment. According to medical records, 34.2% did not adhere to the treatment and 13.9% discontinued the medication due to stock shortages in public pharmacies. Antipsychotics were changed in 11.2% of patients due to lack of therapeutic response and in 5.3% of patients due to adverse reactions to the antipsychotic used (Table 2).

**Table 2:** Characteristics of maintenance treatment for patients followed-up from 2012 to 2017, Brasília—Brazil

Characteristic	Patients, No. (%)
	**(*N* = 625)**
Antipsychotic monotherapy	
Yes	518 (82.9)
No	107 (17.1)
Adherence to treatment	
Yes	411 (65.8)
No	214 (34.2)
Treatment interruption due to lack of medication in public pharmacies	
No	538 (86.1)
Yes	87 (13.9)
Replacement of antipsychotic due to lack of therapeutic response	
No	555 (88.8)
Yes	70 (11.2)
Change of antipsychotic due to ADR^a^	
No	592 (94.7)
Yes	33 (5.3)

^a^Adverse Drug Reactions

Concerning monotherapy patients, the typical antipsychotic group was the most used, 62.9%, and haloperidol, 54.2%, was the most prescribed. The highest readmission rates were for patients using Long-Acting Injections, 70.8%, and typical antipsychotics, 69.9% (Table 3).

**Table 3:** Antipsychotic monotherapy used in the maintenance treatment and readmission rate of patients followed-up from 2012 to 2017, Brasília—Brazil

Antipsychotic group	Antipsychotic	Frequency, No. (%)	Readmission, No. (%)
		***N* = 518**	
Typical (oral)	Chlorpromazine	30 (5.8)	22 (73.3)
	Haloperidol	281 (54.2)	199 (70.8)
	Levomepromazine	11 (2.1)	6 (54.5)
	Thioridazine	4 (0.8)	1 (25.0)
	Total	326 (62.9)	228 (69.9)
Atypical (oral)	Aripiprazole	15 (2.9)	9 (60.0)
	Quetiapine	31 (6.0)	18 (58.1)
	Clozapine	13 (2.5)	7 (53.8)
	Risperidone	66 (12.7)	33 (50.0)
	Olanzapine	19 (3.7)	9 (47.4)
	Total	144 (27.8)	76 (52.8)
**LAIs**^**1**^	**Depot Risperidone**	**6 (1.2)**	**5 (83.3)**
	**Depot Haloperidol**	**42 (8.1)**	**29 (69.0)**
	**Total**	**48 (9.3)**	**34 (70.8)**
Total		518 (100)	338 (65.2)

^1^Long-acting injections antipsychotics

Table 4 (pages 17 and 18) shows the results of the univariate analysis. Tobacco, alcohol, cannabis, or cocaine use, poor adherence to the treatment, interrupted treatment due to lack of medication in public pharmacies, and treatment using typical antipsychotics were statistically associated with an increased risk of readmission. There was a statistically significant influence of substance use on readmission. Non-smokers and alcohol non-users showed a 30.0% reduction in the readmission risk (RR, 0.70; 95% CI, 0.58–0.85) compared to smokers or alcohol users. Those who did not use marijuana or cocaine showed a 28.0% reduction in the readmission risk (RR, 0.72; 95% CI, 0.54–0.97) compared to those who did.

**Table 4:** Results of univariate Cox regression analysis for the rehospitalization of patients in a psychiatric hospital, followed-up from 2012 to 2017. Brasília, Brazil

Factor	β	Relative Risk (95% CI)	*P*-value
Marital status			
With partner	0.00	1.00 [Reference]	NA
No partner	0.10	1.10 (0.92–1.33)	0.295
Sex			
Female	0.00	1.00 [Reference]	NA
Male	0.10	1.11 (0.95–1.29)	0.208
Housing circumstances			
Lives with family members	0.00	1.00 [Reference]	NA
Lives alone	0.06	1.06 (0.72–1.55)	0.768
Lives in a public hostel	0.01	1.01 (0.61–1.65)	0.997
Homeless	0.00	1.11 (0.73–1.68)	0.624
Others Type	-0.59	0.56 (0.18–1.73)	0.310
**Tobacco/Alcohol use**			
Both	0.00	1.00 [Reference]	NA
Tobacco use	-0.12	0.89 (0.72–1.09)	0.264
Alcohol use	-0.25	0.78 (0.59–1.03)	0.083
None	-0.36	0.70 (0.58–0.85)	** < 0.001**
Cannabis/Cocaine use			
Cannabis	0.00	1.00 [Reference]	NA
Cocaine	0.07	1.07 (0.72–1.60)	0.724
Both	0.11	1.12 (0.81–1.55)	0.486
None	-0.32	0.72 (0.54–0.97)	**0.032**
**Presence of lifetime suicide attempt**			
No	0.00	1.00 [Reference]	NA
Yes	0.18	1.20 (0.96–1.50)	0.107
Adherence to Treatment			
Yes	0.00	1.00 [Reference]	NA
No	0.71	2.03 (1.79–2.39)	** < 0.001**
Treatment interruption due to lack of medication in public pharmacies			
Yes	0.00	1.00 [Reference]	NA
No	-0.31	0.73 (0.58–0.92)	**0.006**
Replacement of antipsychotic due to lack of therapeutic response			
No	0.00	1.00 [Reference]	NA
Yes	0.06	1.07 (0.84–1.35)	0.598
Change of antipsychotic due to ADRs ª			
No	0.00	1.00 [Reference]	NA
Yes	0.09	1.09 (0.81–1.46)	0.558
Antipsychotic group used			
Atypical	0.00	1.00 [Reference]	NA
Typical	0.31	1.37 (1.16–1.69)	**0.001**
Antipsychotic used			
Aripiprazole			
No	0.00	1.00 [Reference]	NA
Yes	-0.35	0.70 (0.26–1.88)	0.481
Chlorpromazine			
No	0.00	1.00 [Reference]	NA
Yes	0.09	1.09 (0.81–1.47)	0.575
Clozapine			
No	0.00	1.00 [Reference]	NA
Yes	-0.07	0.93 (0.54–1.61)	0.793
Haloperidol			
No	0.00	1.00 [Reference]	NA
Yes	0.20	1.22 (1.03–1.44)	**0.021**
Levomepromazine			
No	0.00	1.00 [Reference]	NA
Yes	0.33	1.39 (0.82–2.36)	0.225
Olanzapine			
No	0.00	1.00 [Reference]	NA
Yes	-0.25	0.78 (0.49–1.24)	0.289
Quetiapine			
No	0.00	1.00 [Reference]	NA
Yes	-0.26	0.77 (0.54–1.12)	0.171
Risperidone			
No	0.00	1.00 [Reference]	NA
Yes	-0.29	0.75 (0.58–0.96)	**0.024**
Thioridazine			
No	0.00	1.00 [Reference]	NA
Yes	-0.12	1.12 (0.42–3.00)	0.817

*β* Regression coefficient

*95% CI* 95% Confidence Interval

^a^Adverse Drug Reactions

The readmission risk increased 100.3% in individuals who did not adhere to treatment (RR, 2.03; 95% CI, 1.79–2.39). This risk decreased 27.0% in those who did not interrupt the treatment due to lack of medication in the public health system (RR, 0.73; 95% CI, 0.58–0.92) compared to individuals who interrupted for this reason. The risk of readmission of those who used typical antipsychotics increased by 37.0% (RR, 1.37; 95% CI, 1.16–1.69) compared to patients who used atypical antipsychotics. When considering drugs individually, haloperidol’s use increased the risk of readmission by 22.0% (RR, 1.22; 95% CI, 1.03–1.44). On the other hand, the use of risperidone reduced the risk of readmission by 25.0% (RR, 0.75; 95% CI, 0.58–0.96).

Cox multivariate regression model was adjusted from the variables selected in the univariate analysis. The initial model consisted of variables that presented, according to the forward method, a *p*-value of less than 0.250: gender; tobacco use/alcohol use; use of illicit substances; lifetime suicide attempt; treatment adherence; treatment interruption due to lack of medication in public pharmacies; and the group of antipsychotics in use.

There was no evidence of the Cox model’s adequacy using the variable antipsychotics in use, thus opting for the model with the variable group of antipsychotics comprising the group of typical antipsychotics and atypical antipsychotics.

Poor adherence to treatment and the group of typical antipsychotics, whose adjusted relative risk values are shown in Table 5, remained in the model after adjusting the multivariate analysis.

**Table 5:** Final Cox Multivariate Regression Model for readmission of patients in a psychiatric hospital followed-up from 2012 to 2017, Brasília—Brazil

Factor	β	RR adjusted (95% CI)	*P*-value
No adherence to treatment	0.68	1.92 (1.63—2.27)	** < 0.001**
Typical antipsychotic group	0.23	1.25 (1.03—1.52)	**0.023**

*β* Regression coefficient, *RR* Relative Risk, *95% CI* 95% Confidence Interval

By controlling the other variables, the risk of readmission increased by 25.0% (RR, 1.25; 95% CI, 1.03–1.52) when using a typical antipsychotic compared to those who used an atypical one. Individuals who did not adhere to the treatment showed a 92.0% increase (RR, 1.92; 95% CI, 1.63–2.27) in the risk of readmission compared to their adherent peers.

## Discussion

The study shows that most patients (82.9%) used antipsychotic monotherapy and preferably typical antipsychotics (62.9%), with haloperidol as the most prescribed (54.2%) for maintenance treatment. The preference for single antipsychotic follows guidelines that globally endorse the routine practice of antipsychotic monotherapy [31]. However, antipsychotic polytherapy has increased in recent years, despite being more expensive and lacking evidence of its efficacy and safety [32]. Studies show the expanded use of antipsychotic polytherapy in several countries, such as Japan (90%), the United States (58%), East Asian countries (45%), Austria (47%), and Italy (20%) [33]. The preference for non-association of antipsychotics found in the study may be related to the lack of well-defined clinical protocols to support antipsychotic polytherapy and even to the accumulated clinical practice experience developed over the years in the hospital.

Contrary to studies that show a tendency to decrease the prescription of typical antipsychotics and an increase in the prescription of atypical antipsychotics, due to their lower extrapyramidal effects and greater efficiency [4, 21, 34], this study shows a preference for the use of typical antipsychotics in the treatment of the studied population. The frequent choice to prescribe typical antipsychotics, evidenced in our study, may be related to the possible difficulty in accessing atypical antipsychotics in public pharmacies and laboratory tests to monitor patients for adverse reactions they may experience, especially when using clozapine. These hardships make atypical antipsychotics an almost exclusive choice for the treatment of refractory patients.

Although atypical antipsychotics were prescribed the least, patients treated with them had the lowest readmission rate. The higher readmission rate of those treated with LAIs can be explained by the possible greater severity of the patients.

The reasons for discontinuing pharmacological treatment reported in the medical records were evaluated in the study. The main reason for reported interruption was non-adherence to treatment (34.2%), and the non-adherence rate is close to other studies, ranging from 30 to 40% [35–38].

The results differ from the meta-analysis of 46 studies published until December 2017 that evidenced non-adherence to pharmacotherapy for schizophrenia (56%) and bipolar disorder (44%). Another evidence of this meta-analysis was that, besides the patient’s lack of social or family support, clinical factors and the treatment itself and factors related to the health system and services influence non-adherence to treatment [39].

Poor adherence to treatment increases the risk of relapses and, consequently, the risk of rehospitalization of patients with psychotic disorders [40–42], generating a high economic cost to health services [43, 44]. Strategies that include pharmacological treatment and psychosocial interventions, education, and family and social support have effectively increased patient adherence to treatment [45, 46].

The availability of community treatment programs after the first psychotic episode, consisting of a specialized multidisciplinary team for monitoring in the first years of the disease, also reduces the use of hospital services [47]. The use of long-acting injectable antipsychotics is another strategy adopted in recent years to ensure greater adherence to maintenance treatment. However, studies are inconsistent with this alleged assurance [48–56].

Drug-related factors that can also lead to an interruption in the severe mental disorders treatment and contribute to non-adherence were shown in this study. The interruption due to the lack of availability of antipsychotics in public pharmacies was reported in 13.9% of the patients’ medical records, medication change due to the lack of therapeutic response was found in 11.2%, and medication change due to adverse reactions was evidenced in 5.3%.

The shortage of antipsychotics in public pharmacies compromises the treatment of patients with greater social vulnerability. North American studies have shown that the financial cost of antipsychotics for people with psychosis and their family core must be considered a factor associated with non-adherence to treatment. These studies have shown that the greater the patient’s co-payments, the greater the rate of non-adherence to treatment [57, 58].

The universal health coverage system, namely, the Brazilian Unified Health System (SUS), is highly relevant in guaranteeing free treatment for patients with severe mental disorders. However, the lack of a continuous supply of antipsychotics in public pharmacies that are part of the system can interrupt the treatment of the patients in situations of greater social vulnerability.

Changing the antipsychotic in use is one of the pharmacological strategies employed to improve adherence when there is a lack of therapeutic response and the appearance of adverse reactions during treatment. The replacement of one antipsychotic for another requires careful clinical evaluation because new adverse reactions may arise with this intervention, and the disorder may deteriorate due to withdrawal syndromes and loss of effectiveness [59, 60].

Our study found that the risk of readmission was increased by 25.0% for patients treated with typical antipsychotics and by 92.0% for those who did not adhere to maintenance treatment. The results diverge from previous studies showing no difference in the rate and risk of readmission [61, 62] and in the time between readmissions among patients treated with typical and atypical antipsychotics [63]. This this divergence can be explained by the fact that we used a longer follow-up period (we followed-up for 5 years while the other studies followed-up for 1 to 2 years) and because we have had access to hospital and outpatient records, which facilitated our assessment of readmissions better and monitoring the continued use of antipsychotics during outpatient treatment.

The study’s main limitation is that 82.9% of the sample used antipsychotic monotherapy, and 71.0% used a typical antipsychotic. As this study is not a randomized controlled clinical trial, the difference in the risk of readmission between patients treated with typical and atypical antipsychotics may be because most patients used typical antipsychotics in maintenance treatment. The lack of standardized recording of attendance and development in medical records, even in electronic format, also ends up being a limiting factor of the study. The absence of vital sociodemographic and clinical data records may increase information bias in this type of epidemiological study.

## Conclusion

Although typical antipsychotics are preferentially prescribed, statistical analyses indicate greater effectiveness of atypical antipsychotics in treating the maintenance of patients with severe mental disorders. These findings are relevant to assist in decision-making during clinical practice and should be considered in formulating public mental health policies.

## Data Availability

All data generated or analyzed during this study are included in this published article. Data are available on request from the corresponding author.

## Abbreviations

ADR: Adverse Drug Reactions
CI: Confidence Interval
COEP: Research Ethics Committee
DSM-5: Diagnostic and Statistical Manual of Mental Disorders 5^th^ edition
FEPECS-SESDF: Health Sciences Teaching and Research Foundation of the State Health Department of the Federal District
ICD: International Classification of Diseases
LAI: Long-Acting Injections
NIMH: National Institute of Mental Health
R®: R Foundation for Statistical Computing
RR: Relative Risk
WHO: World Health Organization
